# Intrinsically disordered proteins drive membrane curvature

**DOI:** 10.1038/ncomms8875

**Published:** 2015-07-24

**Authors:** David J. Busch, Justin R. Houser, Carl C. Hayden, Michael B. Sherman, Eileen M. Lafer, Jeanne C. Stachowiak

**Affiliations:** 1 Department of Biomedical Engineering, The University of Texas at Austin, 107 W Dean Keeton, Austin, Texas 78712, USA; 2 Sandia National Laboratories, 7011 East Avenue, Livermore, California 94550, USA; 3 Department of Biochemistry and Molecular Biology, University of Texas Medical Branch, 1.224 Medical Research Building, Galveston, Texas 77555, USA; 4 Department of Biochemistry and Center for Biomedical Neuroscience, The University of Texas Health Science Center at San Antonio, UTHSCSA Biochemistry 415B, 7703 Floyd Curl Drive, San Antonio, Texas 78229, USA; 5 Institute for Cellular and Molecular Biology, The University of Texas at Austin, 107 W Dean, Keeton,Texas 78712, USA

## Abstract

Assembly of highly curved membrane structures is essential to cellular physiology. The prevailing view has been that proteins with curvature-promoting structural motifs, such as wedge-like amphipathic helices and crescent-shaped BAR domains, are required for bending membranes. Here we report that intrinsically disordered domains of the endocytic adaptor proteins, Epsin1 and AP180 are highly potent drivers of membrane curvature. This result is unexpected since intrinsically disordered domains lack a well-defined three-dimensional structure. However, *in vitro* measurements of membrane curvature and protein diffusivity demonstrate that the large hydrodynamic radii of these domains generate steric pressure that drives membrane bending. When disordered adaptor domains are expressed as transmembrane cargo in mammalian cells, they are excluded from clathrin-coated pits. We propose that a balance of steric pressure on the two surfaces of the membrane drives this exclusion. These results provide quantitative evidence for the influence of steric pressure on the content and assembly of curved cellular membrane structures.

## Abstract (web summary)

Proteins that bend membranes often contain curvature-promoting structural motifs such as wedges or crescent-shaped domains. Busch *et al.* report that intrinsically disordered domains can also drive membrane curvature and provide evidence that steric pressure driven by protein crowding mediates this effect.

## Graphical abstract

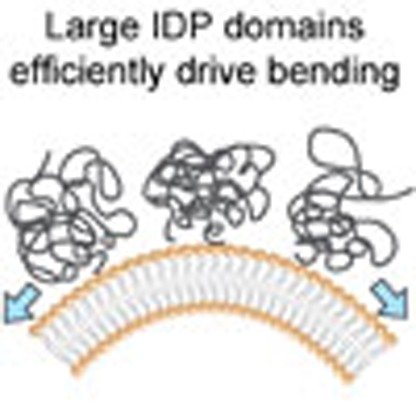

Owing to their critical role in membrane traffic and their accessibility on the plasma membrane surface^1^,^2^, clathrin-coated pits are a model system for studying cellular membrane curvature. Within these highly curved membrane invaginations, transmembrane cargo molecules are linked to the growing clathrin coat by accessory and adaptor proteins, which bind simultaneously to clathrin, cargo and membrane lipids^3^. It is broadly accepted that the clathrin lattice, which assembles spontaneously into highly curved cages^4^,^5^,^6^, and is important for cell growth and viability^7^, plays a key role in driving the curvature of coated pits. However, several studies have demonstrated that the stably folded membrane-interacting domains of adaptors contain structural motifs that can promote membrane curvature, including wedge-like amphipathic helices^8^ and crescent-shaped BAR domains^9^. In contrast, the protein domains that interact with clathrin and other components of the coat, typically the C-terminal domains (CTDs), are often intrinsically disordered^10^,^11^,^12^, including the CTDs of Epsin1, AP180 and CALM (432, 569 and 339 disordered amino acids in rat sequences, respectively), as well as in the clathrin-uncoating protein auxilin (267 disordered amino acids)^13^. Overall, intrinsic disorder is remarkably high in the clathrin endocytic pathway^14^, where it was recently reported that 30% of proteins contain disordered regions of >100 amino acids, including the adaptors above as well as other highly studied proteins such as dynamin, amphiphysin and EPS15 (ref. 15).

Since disordered adaptor domains lack three-dimensional structure and do not interact directly with membranes, they have not been considered as potential drivers of membrane curvature. However, we have recently demonstrated that the pressure generated by collisions among highly crowded proteins of arbitrary structure is sufficient to drive bending^16^,^17^. Specifically, collisions among crowded molecules produce steric pressure^18^,^19^ that favours expansion of the membrane surface^20^,^21^. If this pressure is not balanced by equally crowded molecules on the opposite membrane surface, the resulting imbalance will drive membrane bending towards the more crowded surface. This hypothesis, which has been cited to explain membrane bending during coated vesicle assembly^22^,^23^,^24^,^25^, amyloid formation^26^ and autophagy^27^, suggests that proteins with large hydrodynamic radii should make the largest contribution to membrane crowding, since they occupy the most area on the membrane surface^20^. From this perspective, intrinsically disordered protein domains, which have significantly larger hydrodynamic radii than structured proteins of similar molecular weight^28^, could serve as potent drivers of membrane curvature through crowding. For example, the folded Epsin1 ENTH domain occupies an area of ∼16 nm^2^ on the membrane surface^29^, while the disordered CTDs of Epsin1 and AP180 are expected to occupy 70 and 90 nm^2^, respectively, based on analytical modelling^28^ and experiments^11^. Nonetheless, since the established view has been that structured domains are responsible for curvature generation^1^, the field has overlooked the potential of intrinsically disordered domains to drive membrane curvature.

This work uses a combination of *in vitro* biophysical studies and quantitative experiments in live cells to investigate the ability of intrinsically disordered domains to create steric pressure on membrane surfaces. We find that disordered adaptor domains drive membranes to form highly curved tubules with considerably greater efficiency than structured domains such as the ENTH domain. Further, by investigating the diffusivity of membrane-bound adaptor proteins, we demonstrate that the large hydrodynamic radii of intrinsically disordered domains significantly enhances molecular crowding, explaining their ability to drive bending. To test the impact of molecular crowding on the assembly of coated vesicles, we examine the ability of clathrin-coated pits to internalize cargos that display intrinsically disordered domains on the extracellular plasma membrane surface. These experiments provide quantitative evidence that large, crowded cargo molecules exert substantial steric pressure on clathrin-coated pits, markedly decreasing the quantity of cargo molecules that can be encapsulated, in comparison with a small, globular control cargo.

## Results

### Intrinsically disordered proteins drive membrane curvature

**Figure 1:**
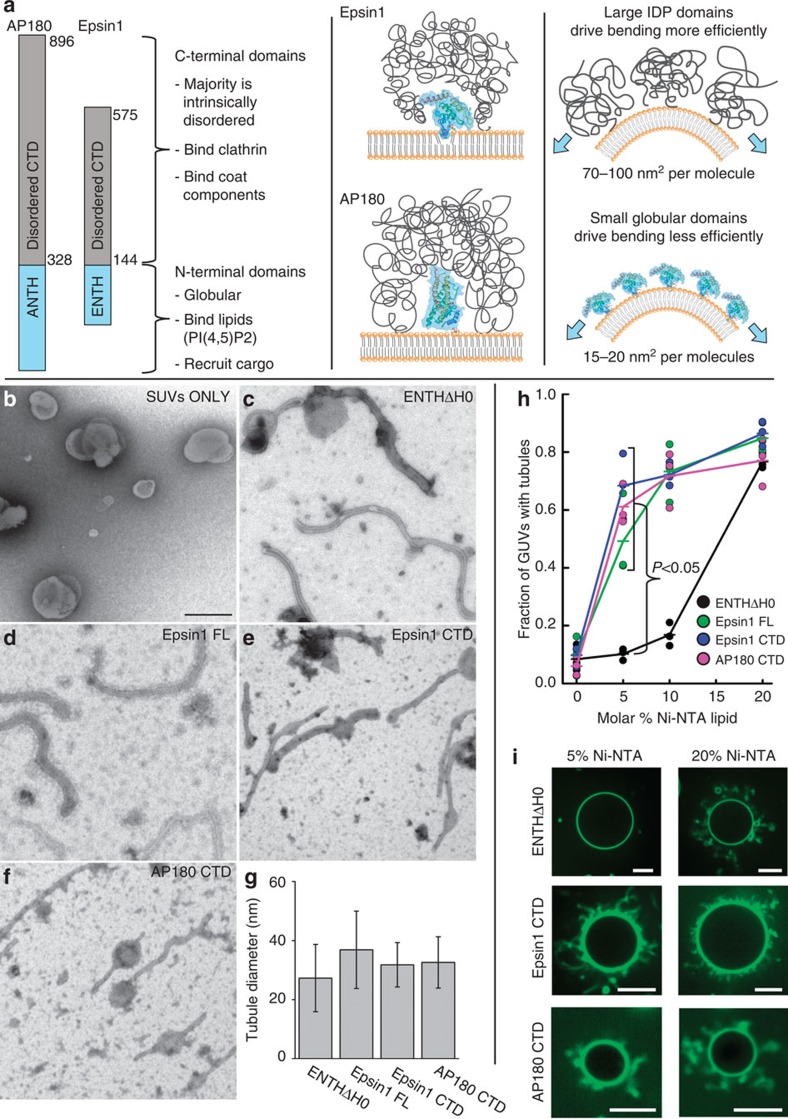
Disordered adaptor domains drive membrane bending efficiently. (**a**) The structure of Epsin1 and AP180 consist of stably folded N-terminal domains that bind to membrane surfaces. In contrast the C-terminal domains of these adaptors are intrinsically disordered polypeptides that make multiple bonds with clathrin, other adaptor proteins and various coat components. These domains have large hydrodynamic radii in comparison with N-terminal domains, suggesting they can occupy larger areas on the membrane surface. (**b**–**f**) Transmission electron micrographs of initially spherical DOPC vesicles (SUVs) containing 20 mol% DOGS-Ni-NTA (**b**) mixed with the following histidine-tagged protein constructs: (**c**) ENTHΔH0, (**d**) Epsin1 FL, (**e**) Epsin1 CTD and (**f**) AP180 CTD. Scale bar is 200 nm and applies to all EM images. (**g**) All constructs drove membrane tubulation, with average tubule diameters in the range of 15–50 nm. Experimental data is from two electron microscope grids per protein. Error bars represent the mean±s.d.; ENTHΔH0, *n*=108 tubules; Epsin1 FL, *n*=255 tubules; Epsin1 CTD, *n*=121 tubules; and AP180 CTD, *n*=81 tubules. (**h**) Fraction of POPC/DOGS-Ni-NTA GUVs that form tubules following binding of histidine-tagged proteins labelled with Atto488 fluorescent dye. Proteins were added at a concentration of 5 μM. Proteins that contained disordered domains (Epsin1 FL, Epsin1 CTD and AP180 CTD) each drove the majority of GUVs to form tubules when 5 mol% DOGS-Ni-NTA was present, while the ENTHΔH0 domain required 20 mol% DOGS-Ni-NTA lipids. *n*=3 independent experiments, >100 GUVs per experiment. ENTHΔH0 tubulates membranes significantly less than each of the other constructs at 5 mol% DOGS-Ni-NTA (Student’s *t*-test, unpaired two-tail, *P*<0.05). (**i**) Representative spinning disc confocal fluorescence images of GUVs containing 5 and 20 mol% DOGS-Ni-NTA after incubation with ENTHΔH0, Epsin1 CTD and AP180 CTD, respectively. Scale bar, 10 μm.

To determine whether intrinsically disordered domains could produce highly curved membrane structures, we incubated his-tagged proteins, including Epsin1 full-length (FL), Epsin1 CTD and AP180 CTD with 200 nm diameter small unilamellar vesicles (SUVs) (Fig. 1b) containing DOGS-Ni-NTA (1,2-dioleoyl-*sn*-glycero-3-[(*N*-(5-amino-1-carboxypentyl)iminodiacetic acid)succinyl] (nickel salt)) lipids, which bind with high affinity to histidine residues. We examined the resulting structures using transmission electron microscopy. As a positive control for membrane tubulation, we also examined vesicles incubated with the ENTH domain, which has been described as the curvature-inducing domain of Epsin1 (ref. 8). Our recent results demonstrated that the ENTH domain has an equal capacity to drive membrane curvature whether the wild-type protein binds to PI(4,5)P_2_-containing membranes using its amphipathic *N*-terminal helix, (helix0), or an engineered histidine tag that replaces the helix binds DOGS-Ni-NTA containing membranes^17^. Specifically, the his-tagged mutant without helix0, abbreviated here as ENTHΔH0, created lipid tubules with the same range of diameters and required the same number of membrane-bound protein molecules per membrane area to drive tubulation as the wild-type ENTH domain. As expected, incubating SUVs with ENTHΔH0 produced highly curved membrane tubules (Fig. 1c)^17^. Similarly, Epsin1 FL, Epsin1 CTD and AP180 CTD were all able to generate highly curved membrane tubules when exposed to SUVs containing the same concentration of DOGS-Ni-NTA lipids, 20 mol% (Fig. 1d–g; Supplementary Fig. 1).

To measure the efficiency of membrane bending by disordered adaptor domains and compare it with the efficiency of bending by the structured ENTH domain, we employed a giant unilamellar vesicle (GUV) tubulation assay. This assay enables rapid screening of large populations of vesicles and dynamic visualization of lipid tubules using fluorescence microscopy. We varied the concentration of DOGS-Ni-NTA lipids to vary the concentration of his-tagged proteins bound to the membrane surface. Consistent with our previous results, ENTHΔH0 was able to tubulate GUV membranes (Fig. 1h–i; Supplementary Fig. 2a)^17^. For all proteins, the fraction of GUVs forming tubules on protein addition increased with increasing DOGS-Ni-NTA concentration (Fig. 1h), even driving some vesicles to collapse at high concentrations (Supplementary Movie 2). However, while 20 mol% DOGS-Ni-NTA was required for strong tubulation by the ENTH domain, Epsin1 FL, Epsin1 CTD and AP180 CTD were all able to strongly tubulate GUVs containing just 5 mol% DOGS-Ni-NTA (Fig. 1h–i; Supplementary Fig. 2b–g). These results demonstrate that disordered adaptor domains are highly efficient drivers of membrane bending. We next sought to determine the specific mechanism of membrane bending by disordered domains.

### IDPs crowd membranes efficiently due to their large radii

**Figure 2:**
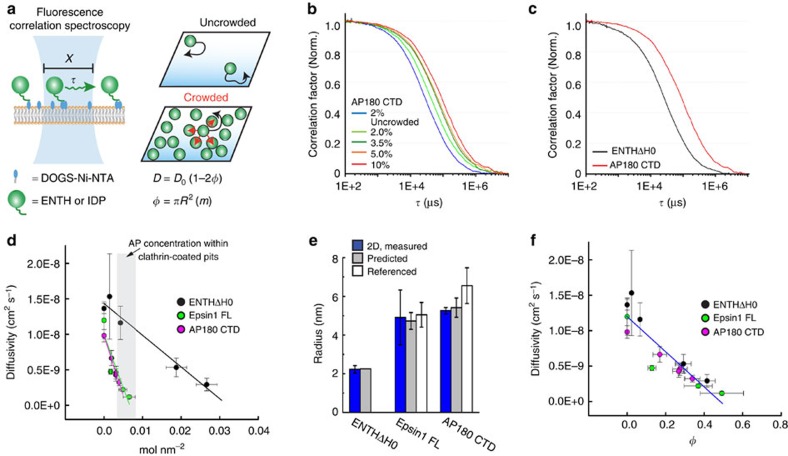
Intrinsically disordered proteins crowd membrane surfaces efficiently. (**a**) Fluorescence correlation spectroscopy (FCS) is used to calculate molecular diffusivity by measuring the time (*τ*) that a fluorescent molecule (green spheres) takes to pass through a laser focal spot. Supported lipid bilayers (SLB) consisting of POPC lipids with increasing concentrations of DOGS-Ni-NTA lipids (blue ovals) were used to modulate the density of membrane-bound, histidine-tagged proteins. Diffusivity (*D*) decreases with molecular coverage, *ϕ* with the relationship *D*=*D*_o_(1–2*ϕ*)*. ϕ* can be calculated as the projected area of a protein, *πR*^2^, multiplied by *m*, the number of molecules per nm^2^. (**b**) Normalized average FCS curves for AP180 CTD on SLB membranes of increasing molar % DOGS-Ni-NTA (2–10 mol%). (**c**) Normalized average FCS curves for ENTHΔH0 (black) compared with AP180 CTD (red). AP180 CTD has a slower time of diffusion despite similar number of molecules per area, ∼4 × 10^−3^ molecules per nm^2^. Experimental *n* values for all FCS data is described in methods. (**d**) Protein diffusivity as a function of the concentration of membrane-bound proteins. Error bars represent the mean±s.d. Experimental *n* values for all membrane coverage data is described in Supplementary Methods. (**e**) Calculated hydrodynamic radii, *R*, from 2D FCS and photon scanning measurements (**b**–**d**), predicted values (ENTH crystal structure or IDP polymer estimates) and referenced gel filtration/analytical centrifugation results^11^. Error bars are the mean±s.d. for 2D measurements, and for referenced measurements. Error bars for predicted sizes represent the range of persistence length values. (**f**) Diffusivity as a function of molecular occupancy, *ϕ*. All proteins collapse onto a single linear curve. Error bars represent the mean±s.d.

Polymer theory^28^ and experimental data^11^ have clearly established that intrinsically disordered proteins have significantly larger hydrodynamic radii than globular proteins of equivalent molecular weight, suggesting that disordered adaptors will crowd membrane surfaces using fewer molecules per membrane area than structured domains. Nonetheless, the deformability of disordered domains makes it difficult to predict how much area they can occupy on a highly crowded membrane surface. Therefore, we measured the number of membrane-bound protein molecules required to crowd the membrane surface for the ENTH domain, Epsin1 FL and AP180 CTD. When molecular crowding increases, molecular diffusivity decreases owing to an increased rate of intermolecular collisions that limit molecular motion^30^,^31^. Accordingly, we would expect that fewer disordered proteins per membrane area are required to drive a decrease in protein diffusivity in comparison with the ENTH domain. To test this hypothesis, we measured protein diffusivity on membrane surfaces as a function of the number of molecules per membrane area. We used fluorescence correlation spectroscopy (FCS) to measure the diffusion time, *τ*, of histidine-tagged protein molecules bound to the surfaces of planar supported lipid bilayers (SLBs) containing DOGS-Ni-NTA (Fig. 2a; Supplementary Fig. 4a–f; Supplementary Movie 3). To vary the number of molecules per membrane area, *m*, membranes included concentrations of DOGS-Ni-NTA lipids ranging from 1 to 10 mol%. To calculate the number of molecules per area, we used quantitative, photon counting scans of fluorescence intensity (see Supplementary Methods). For each of our proteins (ENTHΔH0, Epsin1 FL and AP180 CTD), we observed an increase in the density of membrane-bound molecules with increasing DOGS-Ni-NTA concentration (Supplementary Fig. 3g). As membrane surfaces became more crowded, the diffusion of each protein slowed as expected (Fig. 2a,b; Supplementary Fig. 3a–c). For the disordered proteins, the drop in diffusivity occurred more rapidly with increasing protein concentration. For example, at approximately the same concentration of membrane-bound proteins, AP180 CTD diffused ∼3.5 times more slowly than ENTHΔH0 (Fig. 2c). Under dilute conditions (<0.1% coverage), all of the proteins diffused at similar rates, in the range of 1–1.5 × 10^−8^ cm^2^ s^−1^ (Fig. 2d; Supplementary Fig. 3d–f), consistent with earlier observations that protein diffusivity on membrane surfaces is limited by drag forces within the membrane^32^,^33^. For all the proteins we studied, the decrease in diffusivity with increasing density of membrane-bound proteins was approximately linear (Fig. 2d).

These results are in agreement with a simple, first-order description of the impact of molecular crowding on the diffusivity of non-interacting spherical particles. Here diffusivity is predicted to decrease linearly with increasing fractional molecular occupancy, *ϕ*, according to the relationship, *D*=*D*_0_(1–2*ϕ*), where *D*_0_ is the free diffusivity of non-crowded particles (Fig. 2a)^34^. On a membrane surface, *ϕ* is equivalent to the product of the area that an individual protein covers on the membrane surface and the number of molecules per membrane area, *m*. Approximating the molecular area as *πR*^2^, where *R* is the protein radius, the relationship between diffusivity and the number of molecules per membrane area can be written, *D*=*D*_0_(1–2*πR*^2^*m*). Fitting this relationship to our data yields estimates of the molecular radii, which are in good agreement with the hydrodynamic radii of Epsin1 CTD and AP180 CTD (Fig. 2e) based on theoretical predictions (see Methods), measurements in the literature^11^ and our own dilute solution FCS measurements (Supplementary Fig. 3h). Further, plotting diffusivity as a function of fractional molecular occupancy, *ϕ*, causes data from all proteins to collapse onto a single linear trend with a negative slope of magnitude approximately twice the average uncrowded diffusivity, as predicted by the theory (Fig. 2f). These results demonstrate that the simple theory of crowding among non-interacting spherical particles can reasonably predict the behaviour of crowded proteins on membrane surfaces. Notably, for the highest values of fractional molecular occupancy, *ϕ*, the correlation with diffusivity begins to diverge from the linear trend, likely owing to more complex processes such as molecular aggregation and jamming (Supplementary Fig. 3d,e).

The combined density of all adaptor proteins (AP-2, AP180, Epsin1 and others) within clathrin-coated pits has been estimated to be ∼2–3 per clathrin triskelia^35^,^36^, which is ∼1 adaptor per 60–90 nm^2^ of the membrane surface, based on the physical parameters of clathrin-coated vesicles^37^. Significantly, at this molecular density, ENTHΔH0 displays nearly uncrowded diffusion, while AP180 CTD displays highly crowded diffusion (grey box Fig. 2d). These data suggest that disordered adaptor domains generate substantial steric pressure at concentrations found within clathrin-coated pits (Figs 1a and 2d).

### Crowded IDPs apply pressure within clathrin-coated pits

Having demonstrated that steric pressure among physiological concentrations of disordered adaptor domains is sufficient to drive membrane bending *in vitro*, we sought to determine the impact of steric pressure on the structure and content of clathrin-coated pits in live cells. Loss of the adaptor AP180 results in an increase in the size of synaptic vesicles, implying decreased membrane curvature, as demonstrated by acute perturbation studies in squid^38^, genetic ablation studies in Drosophila^39^ and *Caenorhabditis elegans*^40^, as well as RNA interference studies in cultured mammalian hippocampal neurons^41^. These reports suggest that AP180, and other adaptors of similar structure, play a role in shaping coated vesicles. However, the precise physical impact of adaptor CTDs on vesicle curvature is difficult to differentiate from their biochemical role as recruiters of other coat proteins, which may also drive curvature^42^.

Therefore, to isolate the physical impact of disordered adaptor domains on clathrin-coated pits, we have developed a live-cell assay that is based on the concept of molecular competition. Specifically, we reasoned that whether disordered adaptor domains are capable of creating steric pressure that increases membrane curvature when they are present on the coat side (intracellular surface) of the membrane, and then if the same adaptors were present on the cell’s external surface as cargo molecules, they ought to generate steric pressure that resists internalization by coated pits.

**Figure 3:**
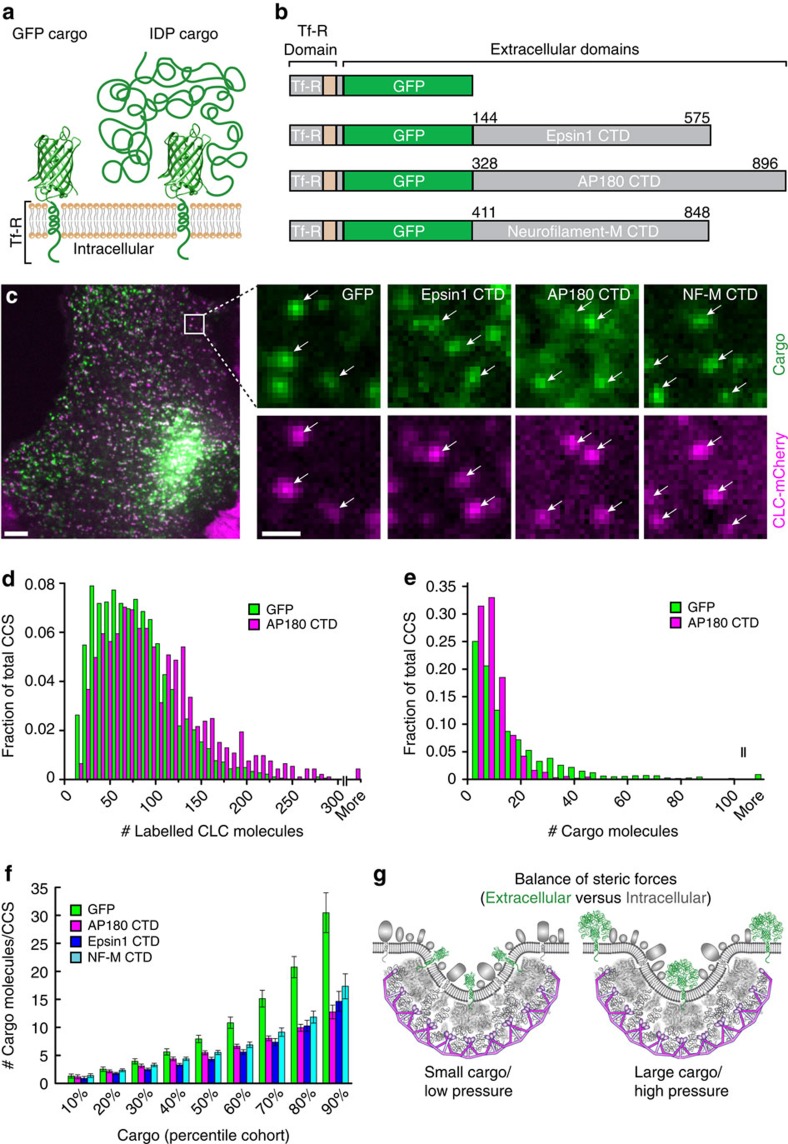
Crowded adaptor CTDs apply physiologically relevant forces to clathrin-coated structures. (**a**) Chimaeric IDP cargo used in cell expression experiments. The control GFP cargo (left) consists of the intracellular and transmembrane domains of the transferrin receptor (amino acids 1–85) containing the YXX*ϕ* internalization motif recognized by the clathrin adaptor AP-2, with an extracellular GFP fluorophore. The IDP proteins Epsin1 CTD, AP180 CTD and neurofilament-M CTD were attached onto the extracellular GFP fluorophore (right) to yield IDP cargo constructs. (**b**) Domain diagrams and the amino-acid lengths for the GFP and IDP cargo. (**c**) Disordered protein cargos were transiently overexpressed in retinal pigmented epithelial (RPE) cells stably expressing mCherry-tagged clathrin light chain (CLC). A single-confocal section of an RPE cell (left) expressing GFP cargo, and insets of the individual channels (right). Arrows mark clathrin-coated structures (CCS) (mCherry), and the colocalized cargo (EGFP). Scale bar for the whole-cell image represents 5 μm. Scale bar for insets represents 1 μm and applies to all images. (**d**) Histogram of number of labelled CLC-mCherry molecules for cells expressing the GFP cargo (green bars, mean±s.d.=74.9±42.4 molecules) compared with cells expressing AP180 CTD cargo (magenta bars, mean±s.d.=95.8±57.9 molecules). (**e**) Histogram of the number of cargo molecules per CCS for cells expressing the GFP cargo (green bars, mean±s.d.=16.2±19.6 molecules) compared with cells expressing the AP180 CTD cargo (magenta bars, mean±s.d.=7.8±7.2 molecules). (**f**) Number of cargo molecules per CCS, displayed as percentile cohorts for each of the cargo constructs (GFP, green bars; AP180 CTD, magenta bars; Epsin1 CTD, blue bars; and NF-M CTD, cyan bars). Data in **d**–**f** is from two dishes of cells per condition representing independent transfections. Experimental *n* values are as follows: GFP, *n*=1825 structures, 17 cells; AP180 CTD, *n*=925 structures, 12 cells; Epsin1 CTD, *n*=1047 structures, 9 cells; and NF-M CTD, *n*=1287 structures, 19 cells. (**g**) Summary model proposing that intrinsically disordered proteins, when crowded, can exert steric pressure that is sufficient to modulate the content of clathrin-coated pits.

To test this idea, we developed chimaeric cargo proteins that display disordered adaptor domains on the surfaces of retinal polarized epithelial (RPE) cells. Specifically, chimeras consisted of the short intracellular and transmembrane domains of transferrin receptor (Tf-R), with the extracellular domain completely replaced by an EGFP domain, followed by the CTDs of either Epsin1 or AP180. The resulting cargo molecules are expected to be highly asymmetric, with disordered adaptor CTDs occupying 70–100 nm^2^ on extracellular plasma membrane surface (Fig. 2e; Fig. 3a,b) and only a few square nanometres on the intracellular surface. For comparison, a control cargo consisted of the same intracellular and transmembrane domains but displayed only an EGFP domain on the plasma membrane surface (Fig. 3a,b). We expect the disordered cargos to have stronger steric interactions with one another and with neighbouring membrane proteins in comparison with the much smaller control cargo. This increase in steric pressure should oppose the internalization of the disordered cargo molecules in clathrin-coated pits. Notably, all chimaeric cargo molecules have the same intracellular and transmembrane domains such that they are biochemically identical from the perspective of the cell’s clathrin machinery. In contrast to knockout studies, this approach leaves clathrin-mediated endocytosis fully intact, and instead sets up a molecular ‘tug of war’ in which the physical impact of adaptor CTDs is judged by measuring their ability to resist incorporation into pits.

Further, to evaluate whether the impact of disordered cargos arises from their large hydrodynamic radii, rather than some more specific characteristic of domains derived from endocytic proteins, we created an additional chimaeric transmembrane cargo consisting of the C-terminal intrinsically disordered CTD of neurofilament-M, a protein with similar size to Epsin1 CTD (438 versus 432 amino acids), yet no role in membrane bending. Neurofilaments are polymeric structural filaments that run the length of neuronal axons. Like bottlebrushes, neurofilaments consist of a structured N-terminal core with large intrinsically disordered CTDs radiating outward along their length^43^. The intrinsically disordered CTDs of neurofilament proteins have been described as an ‘entropic brush,’ and are thought to regulate axonal calibre by occupying volume around the filament, creating steric pressure that repels adjacent filaments^44^. On the basis of its large hydrodynamic radius, we would expect a cargo molecule consisting of the neurofilament-M CTD to increase steric pressure within clathrin-coated pits, similar to those consisting of the CTDs of Epsin1 and AP180. Interestingly, neurofilaments within the axon^45^,^46^ and clathrin coats^35^,^37^ contain similar numbers of disordered domains per volume, based on experimental measurements of the geometry and molecular stoichiometry of each structure.

When transiently expressed in RPE cells stably expressing mCherry-tagged clathrin light chain (CLC-mCherry), spinning disc confocal fluorescence imaging revealed that all of the chimaeric cargos were trafficked to the plasma membrane surface and localized within clathrin-coated structures (Fig. 3c; Supplementary Fig. 5). Analysing our confocal images, we quantified the amount of each cargo within clathrin-coated structures. This analysis utilized publically available software, which has been demonstrated to precisely identify individual clathrin-coated structures on the plasma membrane surface^47^, by fitting a two-dimensional (2D) Gaussian function to the intensity profile associated with each putative clathrin puncta. This approach identifies the subpixel location and intensity of each diffraction-limited puncta with signal amplitude significantly above the local background.

Using this approach to identify clathrin-coated structures and analyse their relative brightness, there was a wide variation in the amount of CLC fluorescence per structure (Fig. 3d; Supplementary Fig. 6d–f), as would be expected for a ‘snapshot’ in time, capturing clathrin structures in all stages of assembly and maturation. Dividing the fluorescence per structure by the single molecule fluorescence of mCherry (see Methods) yielded ∼75 mCherry-labelled CLC molecules per structure for cells expressing the control green fluorescent protein (GFP) cargo. To validate this approach, we used it to analyse SLBs with known concentrations of membrane-bound fluorescent proteins, yielding results in close agreement with independent measurements based on photon counting and FCS (see Methods). Since there are three CLC molecules per triskelia, this measurement suggests there are on average 25 triskelia per structure, which is within the expected range for a growing clathrin-coated pit^37^. As reported recently, labelled CLC molecules are expected to replace a substantial majority of unlabelled endogenous CLC molecules, owing to their overexpression^47^. However, our results may underestimate coat size owing to incorporation of unlabelled endogenous CLC molecules within each structure. We observed a similar, although slightly higher number of CLC molecules per structure among cells expressing the AP180 and Epsin1 CTD cargos (Supplementary Fig. 6j). This increase could arise from an increase in steric pressure that opposes the formation of the smallest, most highly curved pits, although the trend was not maintained for the Nf-M cargo, and could also arise from changes in pit dynamics that were not examined. In contrast, we observed much more significant differences among cells expressing the cargos when we examined their incorporation into clathrin-coated structures.

Once clathrin-coated structures were identified, a 2D Gaussian was fit to the colocalized fluorescence intensity profile of each EGFP cargo puncta. To control for the overall level of cargo expression within cells, we analysed the mean intensity of plasma membrane fluorescence and selected cells with matched levels of expression (Supplementary Fig. 5e). The number of GFP-labelled cargo molecules per structure was quantified exactly as described above for mCherry-labelled CLC (see Methods). We plotted histograms of the number of EGFP-labelled cargo molecules per structure for the control cargo and the AP180 CTD cargo (Fig. 3e; Supplementary Fig. 6a–c), as well as the ratio of cargo to labelled CLC molecules (Supplementary Fig. 6g–i). All histograms indicate a decaying trend in which clathrin-coated structures incorporating a small number of cargo molecules are more frequent than structures incorporating a large number of cargo molecules. However, when disordered cargo molecules were expressed, the distribution shifted to the left, indicating that significantly fewer cargo molecules were incorporated per clathrin-coated structure. The average and median number of chimaeric cargo molecules per structure were: GFP cargo (16, 9), AP180 CTD cargo (8, 6), Epsin1 CTD cargo (8, 5) and Nf-M CTD cargo (10, 6). To highlight the exclusion of disordered cargos from clathrin-coated structures, the data in the histogram is divided into a series of 10 cohorts of equal probability and increasing cargo content. Specifically, all structures were ranked according to the amount of cargo they contained. The bottom 10% were then averaged and plotted as the 10% cohort, the next 10% as the 20% cohort, and so on. Plotting the data in this way enables comparison of events of equal probability. For example, examining the 10% cohort, it is equally probable to find a clathrin-coated structure containing just one copy of any of the cargo molecules in our study. In contrast, examining the 90% cohort reveals that it is equally probable to find a structure that contains 30 GFP cargo molecules as it is to find one that contains only 12–15 disordered cargo molecules.

**Figure 4:**
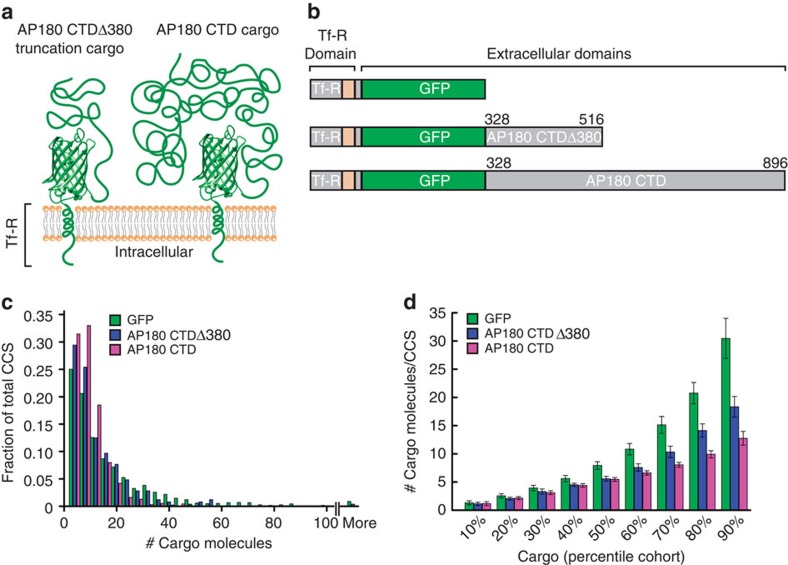
A truncated AP180 CTD cargo molecule demonstrates size dependent exclusion from clathrin-coated structures. A truncated AP180 CTD cargo molecule was made, which only comprises 188 amino acids of the intrinsically disordered C-terminal domain of AP180. (**a**) Cartoon schematic demonstrating that the truncated AP180 C-terminal domain (AP180 CTDΔ380 truncation cargo) should occupy a smaller area on the cell membrane, as compared with the full-length AP180 C-terminal domain (AP180 CTD cargo). (**b**) Diagram of each chimaeric cargo depicting the amino acid lengths. (**c**) Number of cargo molecules per clathrin-coated structure (CCS) in RPE cells expressing GFP cargo, AP180 CTDΔ380 truncation cargo and AP180 CTD cargo. For GFP and AP180 CTD cargos, the histogram data (**c**) and percentile cohort data (**d**) are the same as displayed in Fig. 3f, GFP cargo (green bars, *n*=1825 structures, 17 cells) compared with cells expressing the AP180 CTD cargo (magenta bars, *n*=925 structures, 12 cells). The AP180 CTDΔ380 truncation cargo is incorporated into clathrin-coated structures to intermediate levels (blue bars, *n*=248 structures, 7 cells), further demonstrating the size dependence of cargo exclusion. Values for each percentile cohort represent the mean number of molecules±s.d.

These results suggest that clathrin-coated structures can easily incorporate a few large cargo molecules, but cannot incorporate a large number of disordered cargo, likely owing to increased steric pressure on the membrane surface among the large cargo. Notably, the Nf-M CTD cargo follows the same trend, indicating that it arises from a general physical property of disordered protein domains. As an additional confirmation of this size dependence, we developed a truncated AP180 CTD cargo molecule with approximately two-thirds of the CTD removed which we termed AP180 CTDΔ380 (Fig. 4a,b). This truncated cargo molecule is estimated to occupy a smaller area on the membrane surface. Indeed, this construct was incorporated into clathrin-coated structures to an intermediate degree when compared with the GFP cargo and the FL AP180 CTD cargo molecules (Fig. 4c,d).

## Discussion

Cargo molecules are recruited into clathrin-coated pits by individual adaptor proteins such as AP-2, Epsin1 and AP180. These interactions have relatively low affinities (∼1 μM)^3^,^48^, enabling cargo molecules to transiently associate and dissociate with growing pits and to diffuse from adaptor to adaptor within pits^49^. Our data demonstrates that cargo molecules are excluded from clathrin-coated structures as a function of size. This exclusion effect indicates that when large cargos enter pits they create steric pressure that competes with their affinity for cargo adaptors. This reduced binding favours dissociation of large cargo from pits, reducing steric pressure and thereby enabling curved pits that incorporate a reduced number of large cargos to form.

In contrast, adaptor IDP domains within the coat form multiple bonds with other coat components, leading to a near-permanent affinity for the coated vesicle^50^. Therefore, when adaptor IDP domains become crowded within the coat, the steric pressure they create cannot be relieved by their dissociation. Considering the geometry^37^ and stoichiometry^35^ of an average clathrin-coated vesicle, the number of disordered adaptor and accessory proteins per coated vesicle is around 100, much larger than the typical number of disordered cargo molecules in our studies, <10. The observation that a relatively small number of disordered cargo drives steric exclusion suggests that the greater number of disordered domains in the coat creates substantial steric pressure. The precise mechanisms by which this steric pressure can help to drive membrane invagination during the dynamic assembly of a clathrin-coated pit remain to be revealed, providing an exciting topic for future experiments and theoretical modelling.

These results fit into a larger pattern in which disordered polymers frequently generate steric pressure to achieve diverse cellular goals. As discussed above, the disordered C termini of neurofilaments sterically exclude one another to control axonal calibre^44^. Similarly, disordered phenlalanine–glycine repeats of nucleoporin proteins are thought to provide a steric gating mechanism for the nuclear pore^51^,^52^. Further, the role of disordered polymers in producing steric pressure that prevents nonspecific molecular interactions at biochemical surfaces includes diverse examples from the glycocalyx^53^ to synthetic layers of polyethylene glycol^54^.

While this study has focused on disordered protein domains, other large and highly structured adaptor proteins, such as the tetrameric adaptor protein AP-2 may also generate steric pressure within coats. The structured trunk domains of AP-2 occupy ∼80–100 nm^2^ on the membrane surface^55^, comparable to the disordered adaptor domains. Furthermore, AP-2 is incorporated in pits at approximately a 1:1 stoichiometric ratio with clathrin triskelia^35^, making this protein a major component of pits that could contribute substantially to crowding the membrane surface.

Here we demonstrate that intrinsically disordered domains are a previously unknown class of highly potent membrane bending proteins. By correlating molecular diffusivity with the number of membrane-bound proteins per membrane area, our data demonstrate that the capacity of disordered proteins to bend membranes arises from an enhanced ability to crowd membrane surfaces. Further, live-cell imaging experiments demonstrate that these molecules resist incorporation into clathrin-coated structures when expressed as cargo molecules, owing to steric interactions among them. Consistent with these results, Mettlen *et al.* have shown that overexpression of a chimaeric LDL receptor cargo drives the formation of clathrin-coated structures of low curvature, including large pits and flat clathrin lattices^56^. Similarly, Liu *et al.* observed that attachment of large ligands to cargo ectodomains increased the size of clathrin-coated pits^57^. Further, our results may provide a mechanistic explanation for the recent report by Copic *et al.* that in yeast cells, the COPII coat requires rigid coat components to accommodate large and asymmetric cargo molecules^22^. Finally, our results motivate a re-examination of a recent report by Dannhauser *et al.*^58^, demonstrating that only clathrin and the disordered CTD of Epsin1 are required to reconstitute highly curved clathrin-coated pits. These elegant results were interpreted as a confirmation of clathrin’s capacity to drive membrane curvature, neglecting the potential role of the disordered domain, an effect that our results demonstrate to be highly significant. Taken together our results support a model in which a dynamic balance of steric pressure between coat and cargo proteins modulates the content and curvature of coated membrane vesicles.

## Methods

### Chemical reagents

POPC (1-palmitoyl-2-oleoyl-*sn*-glycero-3-phosphocholine), DOPC (1,2-dioleoyl-*sn*-glycero-3-phosphocholine) and DOGS-Ni-NTA (1,2-dioleoyl-sn-glycero-3-[(N-(5-amino-1-carboxypentyl)iminodiacetic acid)-succinyl] (nickel salt))were purchased from Avanti Polar Lipids (Alabaster, AL, USA). Atto488 fluorescent dye NHS ester was purchased from ATTO-TEC. 4-(2-hydroxyethyl)-1-piperazineethanesulphonic acid (HEPES), phenylmethanesulfonyl fluoride (PMSF), benzamidine hydrochloride, EDTA-free protease inhibitor tablets and TCEP (Tris(2-carboxyethyl)phosphine hydrochloride) were purchased from Sigma-Aldrich (St Louis, MO, USA). Leupeptin and pepstatin were purchased from Roche (Indianapolis, IN, USA). MOPS (3-(*N*-morpholino)propanesulfonic acid), β-mercaptoethanol, Triton X-100, NaCl and PBS tablets were purchased from Fisher Scientific.

### Plasmid constructs

Plasmid constructs used in this study are as follows: ENTHΔH0 was as previously described^17^. The his-Epsin1 FL (Epsin1 FL) and his-Epsin1 CTD constructs, (rat Epsin1, amino acids 144–575; NM_057136) as well as the rat his-AP180 CTD construct (rat AP180, amino acids 328–896; CAA48748) were provided as a kind gifts by Dr Ernst Ungewickell. The constructs GST-6His-Epsin1 CTD (Epsin1 CTD) and GST-6his-AP180 CTD (AP180 CTD) were made by subcloning the PCR amplified CTDs from the above templates into the pGex4T2 vector using incorporated restriction sites EcoRI and XhoI yielding a 27 amino-acid linker (including the 6 his tag) between GST and the CTD. A mouse neurofilament-M plasmid was aquired from Addgene (plasmid #32909; Cambridge, MA, USA), which includes an intrinsically disordered CTD (amino acids 411–848)^59^. The chimaeric Tf-RΔEcto-GFP (GFP cargo) was made by modifying the Tf-R-GFP construct (pEGFP-N1 backbone), kindly provided by Dr Tomas Kirchhausen (Harvard medical school), where the entire Tf-R sequence was excised by digesting with EcoRI and BamHI, and inserting only the PCR amplified intracellular and transmembrane domains of Tf-R adjacent to the GFP fluorophore with EcoRI and BamHI sites. An intermediate construct lacking a stop codon was made by digesting Tf-RΔEcto-GFP with BamHI and NotI to remove GFP, and inserting a mutated PCR amplified GFP with the stop codon (TAA) replaced by a glycine (GGA). For Tf-RΔEcto-GFP Epsin1 CTD (Epsin1 CTD cargo), Tf-RΔEcto-GFP AP180 CTD (AP180 CTD cargo) and Tf-RΔEcto-GFP Neurofilament-M CTD (NF-M CTD cargo), the CTDs of each cargo construct were PCR amplifying using primers containing NotI sites. PCR amplified products were then restriction cloned into NotI sites on the intermediate Tf-R vector described above. The AP180 truncation mutant was cloned using site-directed mutagenesis of Tf-RΔEcto-GFP AP180 CTD plasmid with PCR primers that incorporated a premature stop codon at amino-acid position 516 (A516stop) of the CTD. Following transformation of the DpnI digested reactions, positive clones were selected. The his-mCherry plasmid was acquired from Addgene (plasmid #29722). All constructs were confirmed by DNA sequencing.

### Protein purification

Proteins were purified as previously described^17^,^20^ with the following modifications: proteins were expressed in BL21(DE3) pLysS cells either overnight at 18 °C (ENTHΔH0 and Epsin1 FL), or at 30–37 °C for 3–6 h (Epsin1 CTD and AP180 CTD). Proteins were purified from bacterial extracts either by incubation with DOGS-Ni-NTA agarose in 25 mM HEPES, pH 7.4, 150 mM NaCl, 1 mM PMSF, and 1 mM β‐ME or by glutathione agarose in 0.5 M Tris, 0.1% β‐ME, 4 mM EDTA, 5% glycerol, 1 mM PMSF, 1 mM benzamidine hydrochloride, 7.5 μM pepstatin and 10 μM leupeptin at pH 8.0. Bacteria cells were lysed with 1% Triton X-100 and a horn sonicator at 30% amplitude for 4–6 min in 30-s intervals. After extensive washing, proteins were eluted from DOGS-Ni-NTA or glutathione resins by gradually increasing to a final buffer concentration of 200 mM imidazole or 50 mM glutathione, respectively. Eluted proteins were concentrated and dialyzed for completion with either 25 mM HEPES, pH 7.4, 150 mM NaCl and 1 mM β-mercaptoethanol at 4 °C overnight for DOGS-Ni-NTA agarose purifications, or in 0.5 M Tris, 0.1% β‐ME at 4 °C overnight for glutathione agarose purifications. Proteins were stored as small aliquots at −80 °C.

### Electron microscopy

SUVs were prepared by drying 20 mol% DOGS-Ni-NTA and 80 mol% DOPC lipids into a glass test tube. The lipid film was hydrated with 20 mM MOPS, pH 7.35, 200 mM NaCl. Hydrated films were vortexed and extruded through a 200-nm pore filter (Avanti) to create small vesicles of ∼200-nm diameter. His-tagged proteins were mixed with 1 mM TCEP and incubated with vesicles for 30 min at 37 °C at the indicated protein to DOGS-Ni-NTA lipid molar ratios: ENTHΔH0, 1:10; Epsin1 FL, 1:20–40; Epsin1 CTD, 1:20; AP180 CTD, 1:20. In each case, all DOGS-Ni-NTA-binding sites are expected to be bound by protein. Five microlitres of each solution was added to glow discharged 300 mesh carbon-coated formvar grids and stained with 2% uranyl acetate (Electron Microscopy Sciences; Hatfield, PA, USA). Images were collected from two electron microscope grids per protein condition on a Technai Spirit BioTwin T12 electron microscope (Technai; Hillsboro, OR, USA). Vesicle diameters were measured using ImageJ software.

### Giant unilamellar vesicle preparation

GUVs were electroformed according to published protocols^17^,^60^. Briefly, a lipid composition of POPC with increasing mol% DOGS-Ni-NTA (5–20 mol%) was dried as a lipid film, gently hydrated with 350 mOsm sucrose and electroformed at 55 °C by applying an oscillating potential at 1.4 V peak to peak for 3 h. Vesicles were diluted sixfold in MOPS buffer (20 mM MOPS, pH 7.35; 200 mM NaCl) and incubated with his-tagged proteins containing 5 mM TCEP. All buffers were matched in osmolarity to GUVs using a vapour pressure osmometer. Data were collected from three independent batches of GUVs. More than 100 GUVs per condition were imaged, and the number of GUVs with lipid tubules were counted and calculated as a percent of total GUVs. Lipid tubules were defined as highly dynamic, protein-labelled, extended structures of diffraction-limited diameter.

### Fluorescence microscopy

Spinning disc confocal microscopy (Zeiss Axio Observer Z1 with Yokagawa CSU-X1M) was used to image GUVs and live cells. The laser wavelengths of 488 and 561 nm were used for excitation. Emission filters were centred at 525 nm with a 50-nm width, and 629 nm with a 62-nm width. A triple pass dichroic mirror was used: 405/488/561 nm. The microscope objective used was a Plan-Apochromat 100 × , 1.4 numerical aperture oil immersion objective. GUVs were imaged on a scientific cMOS PCO edge 4.2 camera (PCO AG; Kelheim, Germany). Live-cell experiments were imaged on a cooled (−70 C) EMCCD iXon3 897 camera (Andor Technology; Belfast, UK).

### Supported lipid bilayer preparation

SUVs were prepared by drying down lipid films in clean glass conical tubes. The tubes were put under vacuum for 2–3 h. Then, the lipid film was resuspended in phosphate buffered saline (PBS) to create a 2 mM solution of liposomes. The liposomes were sonicated with a horn sonicator at 75% power for 16 min in 4 min intervals separated by 2 min cooling periods in an ice bath. Ultraclean coverslips were used as the substrate for SLB preparation. Silicone gaskets formed a well on the coverslip surface. PBS was used to initially hydrate the coverslip; then a 2 mM solution of SUVs was pipetted onto the coverslip, followed by a 15 min incubation period during which the SLB formed. The SLB was washed repeatedly with PBS to remove SUVs left in solution. After washing, protein solution was pipetted onto the SLB and incubated for the experiments described below.

### Fluorescence correlation spectroscopy

SUVs consisting of POPC lipids and varying percentages of DOGS-Ni-NTA lipids were used to prepare SLBs. A small concentration of Atto488-labelled protein (2–8 nM depending on the protein and mol% DOGS-Ni-NTA) was prebound to the SLB surface for 30 min for ENTHΔH0 and Epsin1 FL, and 60 min for AP180 CTD. The SLB was washed to remove excess fluorescent protein. Then, a 2 μM solution of unlabelled protein was added to the SLB to create a crowded membrane surface (20-min incubation for Epsin1 FL and ENTHΔH0, 60-min incubation for AP180). Point FCS measurements were acquired using a custom-built time-correlated single-photon counting confocal microscope that has been described previously^20^. FCS curves were collected for 250–500 s using autocorrelation through Becker and Hickl data acquisition software. Experimental *n* values for FCS curves are as follows: for ENTHΔH0, *n*=9 curves, 2 SLBs (2% DOGS-Ni-NTA, uncrowded); *n*=14 curves, 2 SLBs (1% DOGS-Ni-NTA); *n*=12 curves, 2 SLBs (2% DOGS-Ni-NTA); *n*=19 curves, 3 SLBs (5% DOGS-Ni-NTA); *n*=15 curves, 2 SLBs (7.5% DOGS-Ni-NTA); and *n*=13 curves, 2 SLBs (10% DOGS-Ni-NTA); and for Epsin1 FL, *n*=10 curves, 2 SLBs (2% DOGS-Ni-NTA, uncrowded); *n*=6 curves, 1 SLB (1% DOGS-Ni-NTA); *n*=11 curves, 2 SLBs (2% DOGS-Ni-NTA); *n*=9 curves, 2 SLBs (3.5% DOGS-Ni-NTA); *n*=7 curves, 2 SLBs (5% DOGS-Ni-NTA); for AP180 CTD, *n*=13 curves, 2 SLBs (2% DOGS-Ni-NTA, uncrowded); *n*=12 curves, 2 SLBs (2% DOGS-Ni-NTA); *n*=12 curves, 2 SLBs (3.5% DOGS-Ni-NTA); *n*=17 curves, 3 SLBs (5% DOGS-Ni-NTA); and *n*=15 curves, 2 SLBs (10% DOGS-Ni-NTA). The autocorrelations obtained were fit with the standard 2D autocorrelation function:




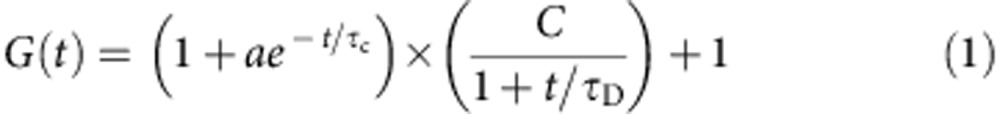




where *C* is 1/*N*_p_, and *N*_p_ is the average number of diffusing proteins in the excitation region. *τ*_D_ is the diffusion time at which the autocorrelation function reaches one-half its initial amplitude. Corrections for short time processes such as intersystem crossing are included in *a* and *τ*_c_, which are held constant in the fitting. They have little effect on fitting the longer time processes of interest here. The autocorrelation curves were fit with a custom nonlinear fitting program using the Levenberg–Marquardt algorithm implemented in ALGLIB (Sergey Bochkanov, www.alglib.net). In crowded Epsin1 FL data sets there was a contribution from labelled protein diffusing in the buffer leading to a fast diffusion component. These data sets were fit using a pseudo three-dimensional (3D) term, which has the form of a 2D autocorrelation term:




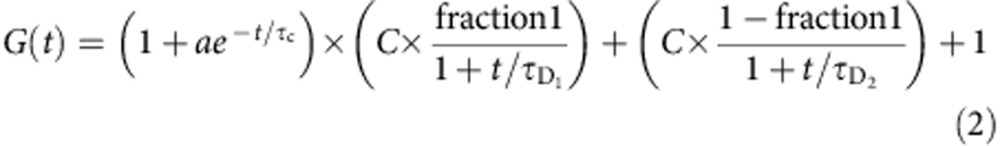




where fraction1 is the fraction diffusing with the fixed diffusion time of *τ*_D1_ for the fast component, 0.8 ms, corresponding to the approximate solution phase diffusion time for this protein. This approximation is appropriate because a 3D term approaches the form of a 2D term in the limit that the *z* axis of the focal volume is long. The larger *τ*_D2_ corresponding to membrane diffusion is reported.

### Estimation of hydrodynamic radii based on polymer theory

A first-order estimate of the hydrodynamic radius, *R*_g_, of a random, self-avoiding polymer chain can be calculated from the following equation, 
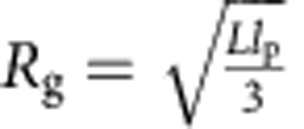
, in which *l*_p_ is the chain persistence length and *L* is the total length of the fully extended polymer chain. The average persistence length of an intrinsically disordered polypeptide has been measured in the range of 0.4±0.07 nm (ref. 28). The total lengths of the disordered domains considered in this study can be approximated by multiplying the length of a single amino acid (that is, the distance between two alpha carbon atoms), 0.38 nm (ref. 61), by the total number of amino acids in the chain, yielding the following chain length values: AP180 CTD (569 amino acids, 216 nm), Epsin1 CTD (432 amino acids, 164 nm) and neurofilament-M CTD (438 and 166 nm). Using these values, the equation above yields the following approximate hydrodynamic radii: AP180 CTD (5.4±0.5 nm), Epsin1 CTD (4.7±0.4 nm) and neurofilament-M CTD (4.7±0.4 nm), where the error arises from the range of persistence length values. Assuming each molecule occupies a sphere of radius *R*_g_, the area it occupies on the membrane surface can be approximated as, 
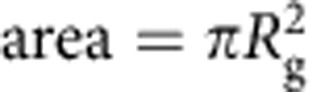
, yielding the following estimates of occupied area: AP180 CTD (91±16 nm^2^), Epsin1 CTD (69±12 nm^2^) and neurofilament-M CTD (70±12 nm^2^).

### Estimation of hydrodynamic radii from 3D diffusion times

Fluorescence correlation microscopy was used to quantify the diffusivity of intrinsically disordered protein domains in dilute, 3D solution. The Stokes-Einstein theory for diffusion of spherical particles in dilute viscous solution can be used to derive first-order estimates of hydrodynamic radius from the measured values of diffusivity. The Stokes-Einstein equation states that 
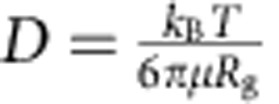
, where *D* represents diffusivity, *μ* represents the solution viscosity, *R*_g_ represents the radius of gyration of the protein molecule, *k*_B_ represents Boltzmann’s constant and *T* represents temperature. We estimated *μ* as approximately the viscosity of water at 17 °C and atmospheric pressure. Rhodamine 6G was used as a reference molecule for solution FCS, where its diffusivity has been independently reported, 3.3 × 10^−6^ cm^2^ s^−1^ (ref. 62), to relate the time of diffusion for Rhodamine 6G as a ratio for the time of diffusion for each of our proteins. The diffusivities were thus determined from time of diffusion measurements as follows: ENTHΔH0 (7.4 × 10^−7^ cm^2^ s^−1^), AP180 CTD (2.4 × 10^−7^ cm^2^ s^−1^) and Epsin1 FL (4.4 × 10^−7^ cm^2^ s^−1^). With these inputs, the Stokes-Einstein equation yields the following estimates of *R*_g_: ENTHΔH0 (2.7 nm), AP180 CTD (8.4 nm) and Epsin1 FL (4.5 nm), in reasonable agreement with estimates based on polymer theory and the crystal structure of the ENTH domain.

### Calibration of the brightness of EGFP and mCherry molecules

To estimate the number of cargo molecules per clathrin coated structure, we divided the brightness of puncta in the cargo image by the measured brightness of puncta within a control sample that consisted of individual EGFP molecules adhered to a coverslip surface. To determine the brightness of individual fluorescent molecules, a dilute solution (∼70 pM) of EGFP was added to an ultraclean coverslip hydrated with PBS. Images of the coverslip surface were acquired with the same laser power and camera gain settings used in all live-cell imaging experiments. A manual approach was first used, where individual fluorescent puncta corresponding to single EGFP molecules were selected from images as diffraction-limited structures of uniform brightness that were sparse on the coverslip. A Gaussian function was fit to the pixel intensity profile of lines drawn through each puncta. To determine the amplitude of the Gaussian function associated with individual molecules, the background fluorescence signal was subtracted. To validate this approach, we also used the particle detection software (described below for clathrin-coated pit detection), to measure the fluorescence amplitude of Gaussian functions fit to individual EGFP molecules. The Gaussian fluorescence amplitude from individual EGFP molecules was used to determine the number of EGFP molecules in diffraction-limited puncta corresponding to clathrin pits, simply by dividing the amplitude for the pit cargo by the amplitude of a single EGFP. This approach extrapolates from the brightness of a single EGFP molecule to determine the number of EGFP-labelled cargo molecules per pit. Since some pits contained 30 or more cargo molecules, it is important to validate this approach independently for large number of EGFP molecules. Therefore, we created SLBs with a density of EGFP molecules diffusing freely on their surfaces, in the upper concentration range of cargo molecules that are incorporated into clathrin-coated pits (at least 10 molecules per confocal focus). Scans recorded the number of photons per area per time from multiple locations over the SLB surface. These values were divided by the corresponding values for single EGFP molecules to determine the number of EGFP molecules bound to the membrane surface per area. Then, the SLB was imaged by spinning disc confocal microscopy to acquire the average fluorescence intensity. The average intensity was divided by the number of molecules per area determined by the photon scans, to provide an independent calibration of the single molecule brightness for EGFP at high density. The two methods of calibration, (i) photon counting and (ii) extrapolation from the intensity of a single EGFP molecule, agreed within ∼20% error when used to determine the number of EGFP molecules on the SLB surface. This test validates our approach of extrapolating from the brightness of a single EGFP molecule to determine the number of cargo molecules within clathrin-coated structures in our live-cell imaging experiments. A similar approach was followed to measure the single molecule fluorescence of mCherry to determine the number of labelled CLC molecules within clathrin structures.

### Cell culture and transfection

Human RPE cells stably expressing mCherry-tagged CLCs were a kind gift from Dr Allen Liu (University of Michigan) and Dr Sandra Schmid (UT Southwestern). RPE cells were grown in 1:1 F-12: DMEM media supplemented with 10% FBS, Pen/Strep/L-glutamine (100 units per ml; 100 μg ml^−1^; 300 μg ml^−1^, respectively) and incubated at 37 °C with 5% CO_2_. Cells were grown for 24 h on 35-mm collagen-coated glass bottom dishes (Matek) before being transfected with 1 μg chimaeric GFP cargo plasmid DNA using Fugene transfection reagent (Promega) and imaged ∼16 h after transfection. Data were collected from two dishes of cells per chimaeric cargo representing independent transfections. Spinning disc confocal images were acquired for clathrin (mCherry) and cargo (EGFP) channels at the plasma membrane surface. Cells were selected based on their level of expression, and only cells with matched levels of background expression were included in the analysis. A 20 × 20-μm crop from the image of each cell was used for analysis. Individual clathrin-coated structures were detected and analysed using particle detection software developed previously and made publically available^47^. Clathrin-coated structures were detected by fitting a 2D Gaussian function to the intensity profile of each putative clathrin-coated structure, where the s.d. (*σ*) of the Gaussian was determined from the point spread function of the microscope system. Signals were reported as valid if the puncta was diffraction-limited and had an amplitude significantly above the local fluorescence background signal as validated previously^47^. Using the subpixel locations of each clathrin puncta, a 2D Gaussian function was then fit to the fluorescence signals within the corresponding regions of the EGFP (cargo) image. Puncta in the cargo image were considered valid if their locations were within 3 s.d. of the location of a corresponding structure in the clathrin signal. To ensure that only puncta with sufficient signal were analysed, we only included puncta with a cargo intensity 1 s.d. above the local background fluorescence signal.

## Additional information

**How to cite this article:** Busch, D. J. *et al.* Intrinsically disordered proteins drive membrane curvature. *Nat. Commun.* 6:7875 doi: 10.1038/ncomms8875 (2015).

## Supplementary Material

Supplementary Figures and Supplementary Methods Supplementary Figures 1-6 and Supplementary Methods

Supplementary Movie 1 Lipid tubules formed on the surface of a giant unilamellar vesicle (5 mol. % DOGS-Ni-NTA lipids / 95 mol. % POPC lipids) by membrane binding of 5 μM Epsin1 CTD-Atto 488. Tubules fluctuate dynamically. The scale bar is 10 μm long. The frames are approximately 200 ms apart. The movie plays in approximately real time.

Supplementary Movie 2 Giant unilamellar vesicles frequently collapsed owing to extensive tabulation of the membrane (20 mol. % DOGS-Ni-NTA lipids / 80 mol. % POPC lipids) when incubated with 5 μM Epsin1 FL. The scale bar is 10 μm long. The frames are approximately 200 ms apart. The movie plays in approximately real time.

Supplementary Movie 3 Diffusion of his-tagged GFP (5 nM) on a Supported Lipid Bilayer (SLB) containing 5 mol. % DOGS-Ni-NTA demonstrates free diffusion of individual molecules on the membrane surface. Images were collected by spinning disc confocal microscopy using 30 ms exposure time. Movie plays at 5 frames per second. Scale bar is 10 μM.
