# Supplementary material for: Intrinsically disordered proteins drive membrane curvature
Source: Nat Commun. 2015 Jul 24;6:7875. doi: 10.1038/ncomms8875 (PMC4515776; doi:10.1038/ncomms8875)
Supplement: Supplementary Figures and Supplementary Methods — Supplementary Figures 1-6 and Supplementary Methods [file ncomms8875-s1.pdf]

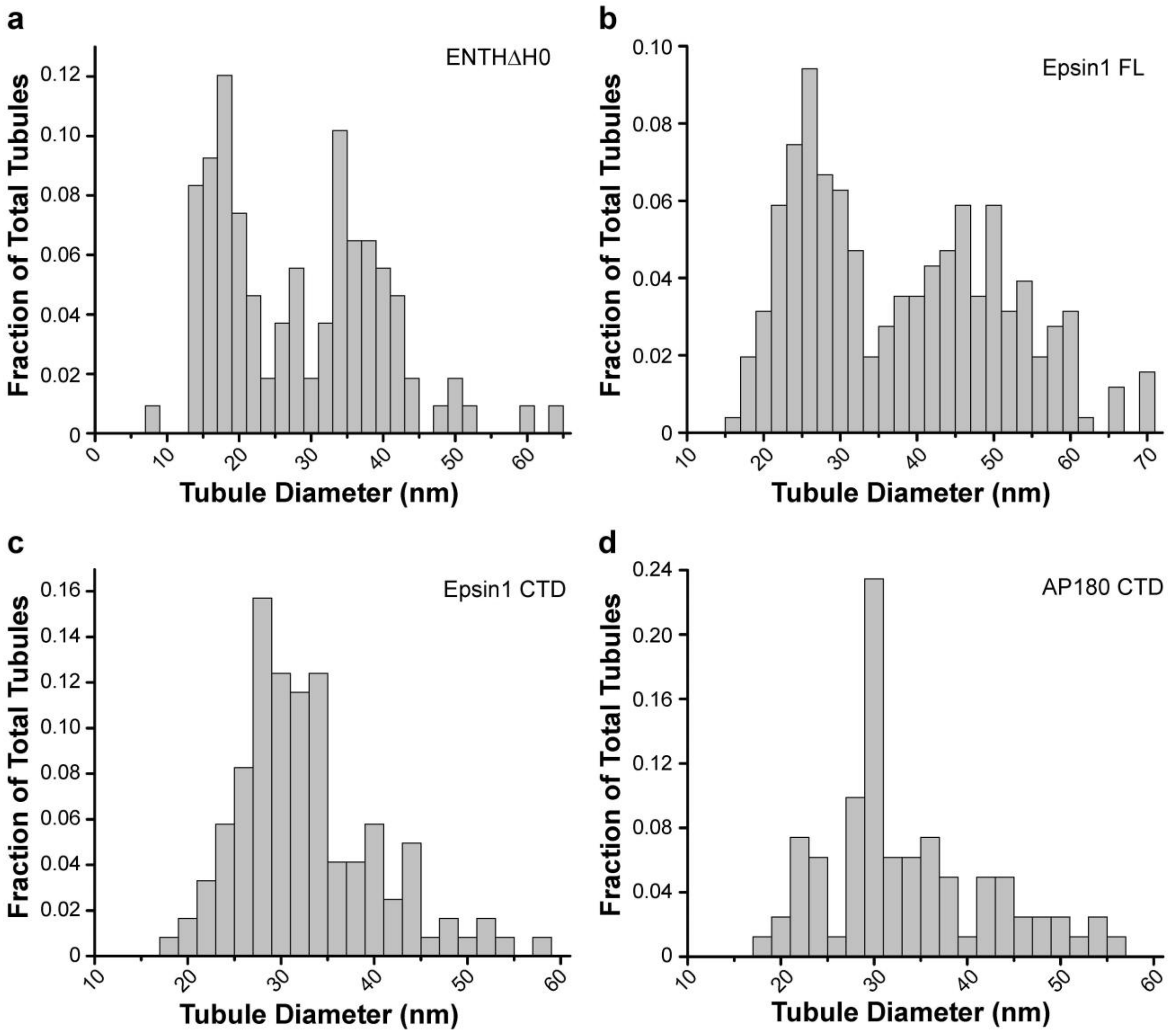

Supplementary Figure 1. Frequency distribution of SUV tubule diameters measured by transmission electron microscopy. Diameter distribution of SUV tubules generated with (a) 20 µM ENTHΔH0, N = 108 tubules. (b) 5-10 µM Epsin1 FL, N = 255 tubules. (c) 10 µM Epsin1 CTD, N = 121 tubules. (d) 10 µM AP180 CTD, N = 81 tubules. SUVs were composed of DOPC with 20 mol. % DOGS-Ni-NTA extruded to 200 nm diameters. Proteins were incubated for 30 minutes at 37°C before depositing and staining on carbon TEM grids. Data was acquired from 2 EM grids per protein condition.

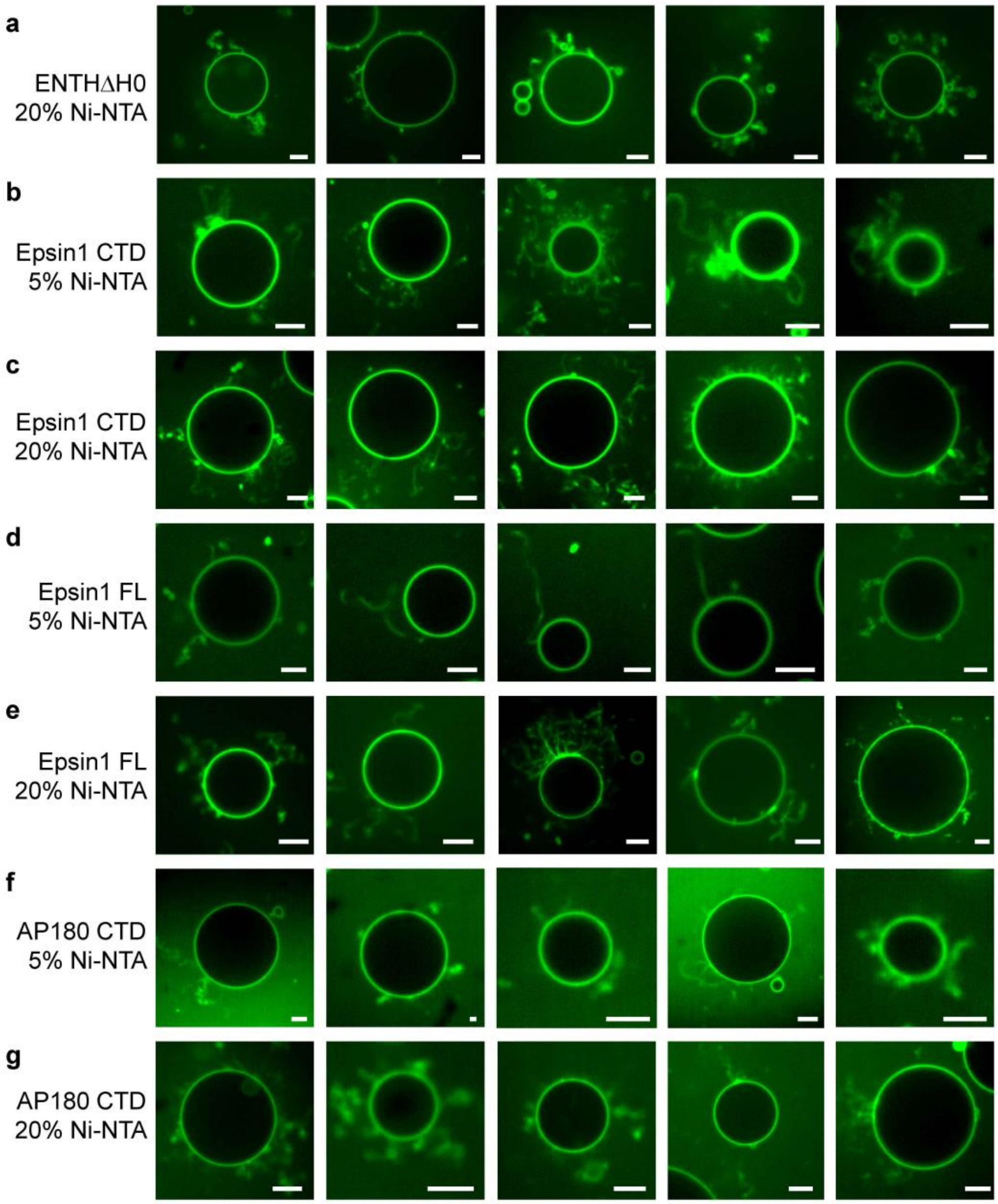

Supplementary Figure 2. Tubulation of GUVs by binding of histidine-tagged proteins labeled with Atto 488 fluorescent dye. Proteins were added at a concentration of 5 μM. GUVs were incubated for 10 minutes prior to observation. Lipid tubules were defined as highly dynamic, protein-labeled, extended structures of diffraction limited diameter. The figure shows 5 example images from each of the following conditions under which frequent membrane tubulation was observed (see Figure 1h): (row a) ENTHΔH0 binding to vesicles containing 5 mol. % DOGS-Ni-NTA lipids, (row b) Epsin1 FL binding to vesicles containing 5 mol. % DOGS-Ni-NTA lipids, (row c) Epsin1 FL binding to vesicles containing 20 mol. % DOGS-Ni-NTA lipids, (row d) Epsin1 CTD binding to vesicles containing 5 mol. % DOGS-Ni-NTA lipids, (row e) Epsin1 CTD binding to vesicles containing 20 mol. % DOGS-Ni-NTA lipids, (row f) AP180 CTD binding to vesicles containing 5 mol. % DOGS-Ni-NTA lipids, (row g) AP180 CTD binding to vesicles containing 20 mol. % DOGS-Ni-NTA lipids.

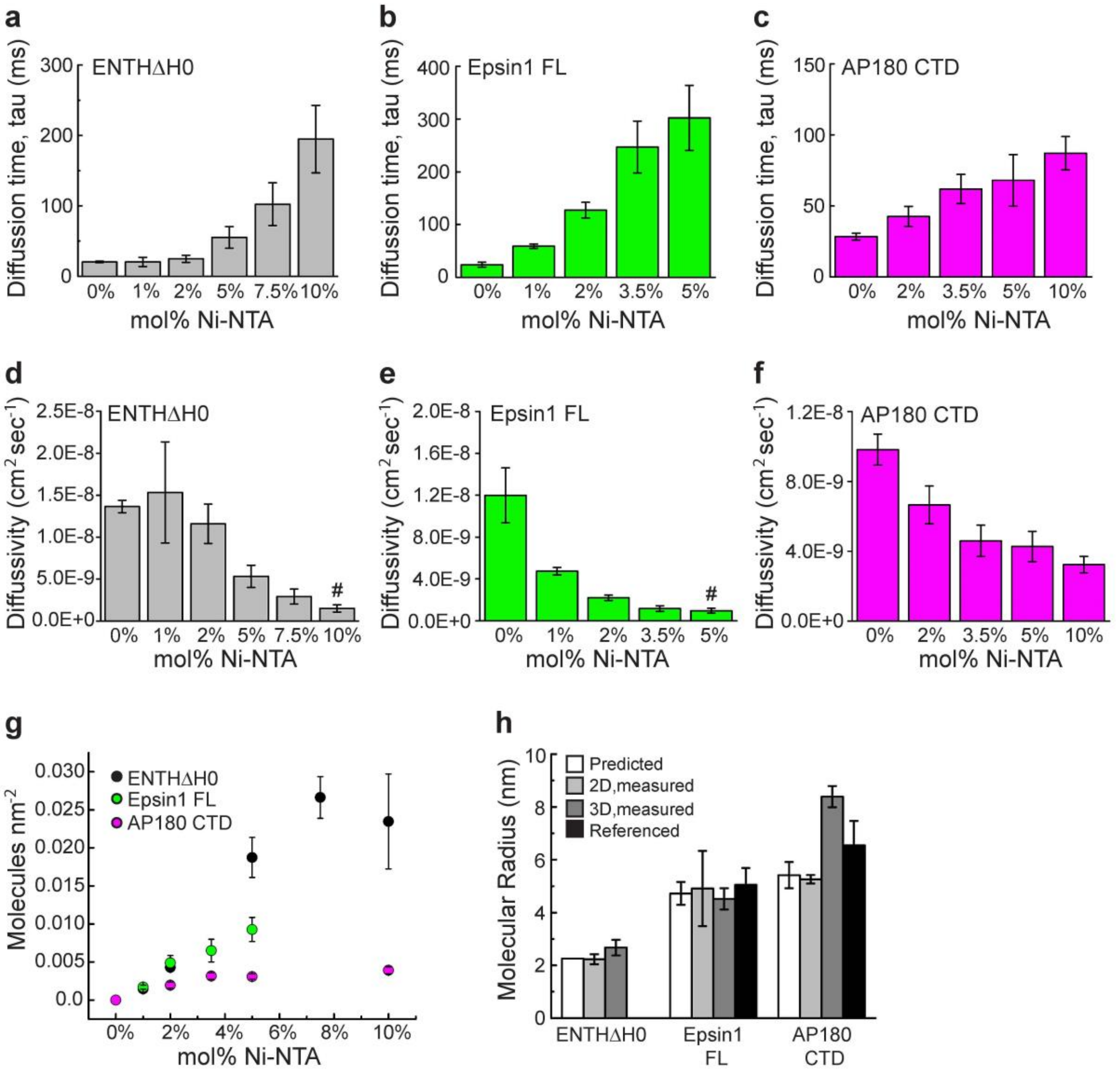

Supplementary Figure 3. Diffusivity decreases with increasing number of molecules per area of membrane surface. Supported lipid bilayers composed of POPC lipids and increasing molar % of the binding lipid DOGS-Ni-NTA are used to measure the molecular diffusivity of bound disordered proteins. Time of diffusion, tau, measured with FCS is plotted as a function of DOGS-Ni-NTA lipid for (a) ENTHΔH0, (b) Epsin1 FL, and (c) AP180 CTD. Diffusivities (cm^2 sec^{-1}), calculated from focal diameter and tau, are plotted for (d) ENTHΔH0, (e) Epsin1 FL, and (f) AP180 CTD. (g) Number of molecules per area of SLB membranes is plotted as a function of molar % DOGS-Ni-NTA for ENTHΔH0 (black circles), Epsin1 FL (green circles), and AP180 CTD (magenta circles). Experimental n values for data in a-g are described in the methods. All values represent the mean ± s.d. (h) Calculated hydrodynamic radii, R, from predicted values (ENTH crystal structure or IDP polymer estimates), 2D FCS and photon scan measurements (Figure 2b-d), 3D solution FCS measurements and referenced gel filtration/analytical centrifugation results. For 3D solution FCS measurements: ENTHΔH0, n = 3 FCS curves; Epsin1 FL, n = 8 FCS curves; AP180 CTD, n = 6 FCS curves. Error bars for 2D, 3D, and referenced measurements are the mean ± s.d. Error bars for predicted sizes represent the range of persistence length values.

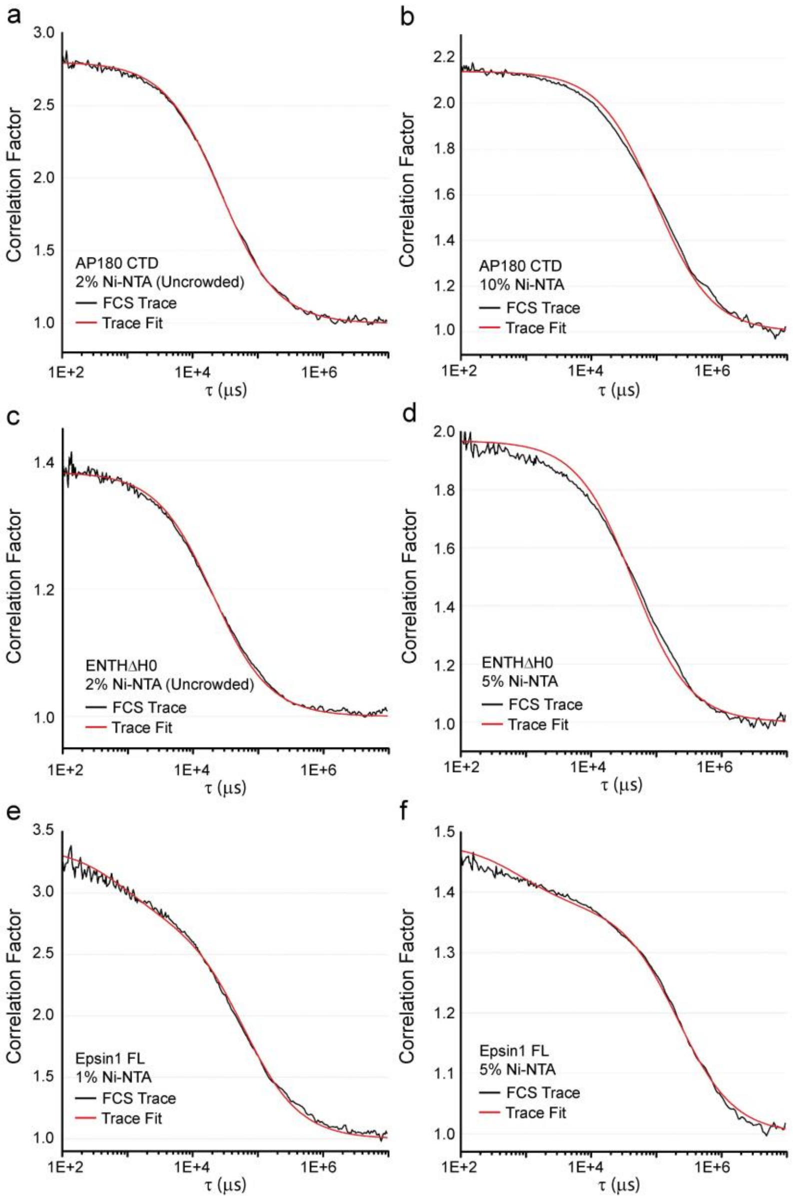

Supplementary Figure 4. FCS autocorrelation traces are well fit by one or two component equations. Example raw FCS autocorrelation curves (black lines) for purified proteins bound to DOGS-Ni-NTA containing membranes plotted with fit data from autocorrelation functions (red lines). (a-b) Raw autocorrelation curves for AP180 CTD bound to a SLB surface at (a) low protein densities on 2% DOGS-Ni-NTA membranes (uncrowded), compared to (b) high protein densities on a 10% DOGS-Ni-NTA membrane. (c-d) Raw autocorrelation curves for ENTHΔH0 protein bound at (c) low protein densities on a 2% DOGS-Ni-NTA membrane (uncrowded), compared to (d) high protein densities on a 5% DOGS-Ni-NTA membrane. AP180 CTD and ENTHΔH0 data were fit using autocorrelation functions that included a single 2D diffusion constant. (e-f) Raw autocorrelation curves of Epsin1 FL protein bound to a SLB membrane at (e) low protein concentrations on a 1% DOGS-Ni-NTA membrane, compared to (f) high protein concentrations on a 5% DOGS-Ni_NTA membrane. As described in the methods, Epsin1 FL required a two component fit to account for the faster diffusion of molecules in solution. All data was well fit using the equations described in methods.

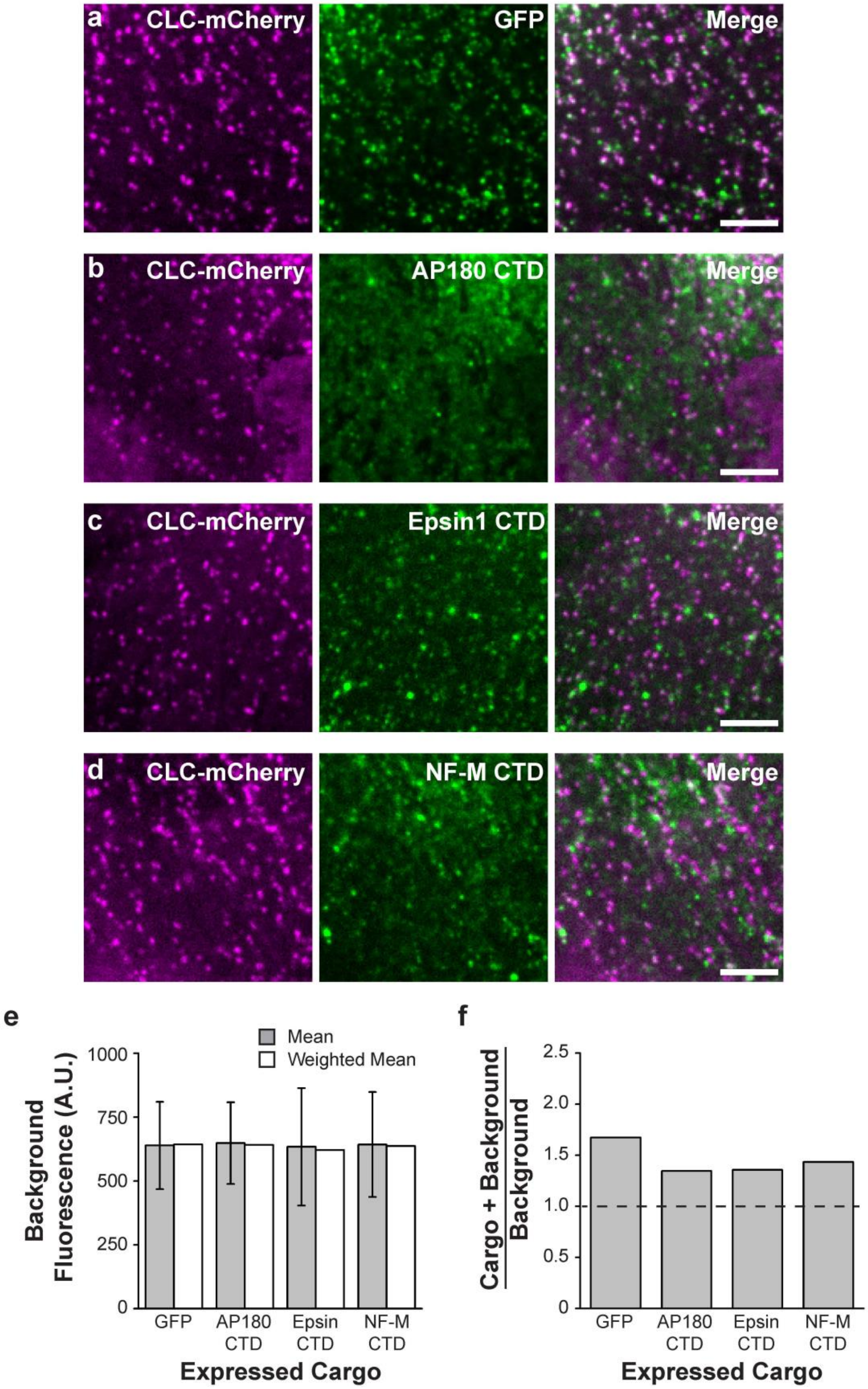

a
CLC-mCherry
GFP
Merge
b
CLC-mCherry
AP180 CTD
Merge
c
CLC-mCherry
Epsin1 CTD
Merge
d
CLC-mCherry
NF-M CTD
Merge
e
Mean
Weighted Mean
Background Fluorescence (A.U.)
1000
750
500
250
0
GFP
AP180 CTD
Epsin CTD
NF-M CTD
Expressed Cargo
f
Cargo + Background / Background
2.5
2.0
1.5
1.0
0.5
0
GFP
AP180 CTD
Epsin CTD
NF-M CTD
Expressed Cargo

Supplementary Figure 5. GFP and IDP cargo colocalize to clathrin-coated structuress and are expressed to equivalent levels. 20 μm x 20 μm cropped images of confocal sections of retinal pigmented epithelial (RPE) cells stably expressing mCherry-tagged clathrin light chain (magenta). The chimeric cargo molecules (a) GFP cargo, (b) AP180 CTD cargo (c) Epsin1 CTD cargo (d) NF-M CTD cargo (green) were transiently expressed in RPE cells. Images represent the cells from which figure 3c cropped images were selected. Scale bars represent 5 μm and apply to all images. (e) Background expression levels of cells used in Figure 3 cell analysis were approximately equal for all chimeric cargo molecules. Values reported are the mean background per clathrin-coated structure ± s.d. (gray bars), and the weighted mean per pit relative to total structures counted per cell (white bars). (f) The sum of cargo and background fluorescence is divided by background fluorescence to provide a ratio of signal to background for each cargo puncta. Bars represent the mean for each cargo molecule demonstrating that the fluorescence intensity of each cargo puncta is above the cell's background fluorescence. Since the location of cargo puncta is determined by the position of each clathrin structure (see methods), this plot also provides a measure of colocalization between cargo and clathrin signals used in the analysis. Experimental n values are as follows: GFP control cargo: n = 1825 structures, 17 cells; AP180 CTD cargo: n = 925 structures, 12 cells; Epsin1 CTD cargo: n = 1047, structures, 9 cells; NF-M CTD: n = 1287 structures, 19 cells. Data was acquired from 2 dishes of cells per condition representing independent transfections.

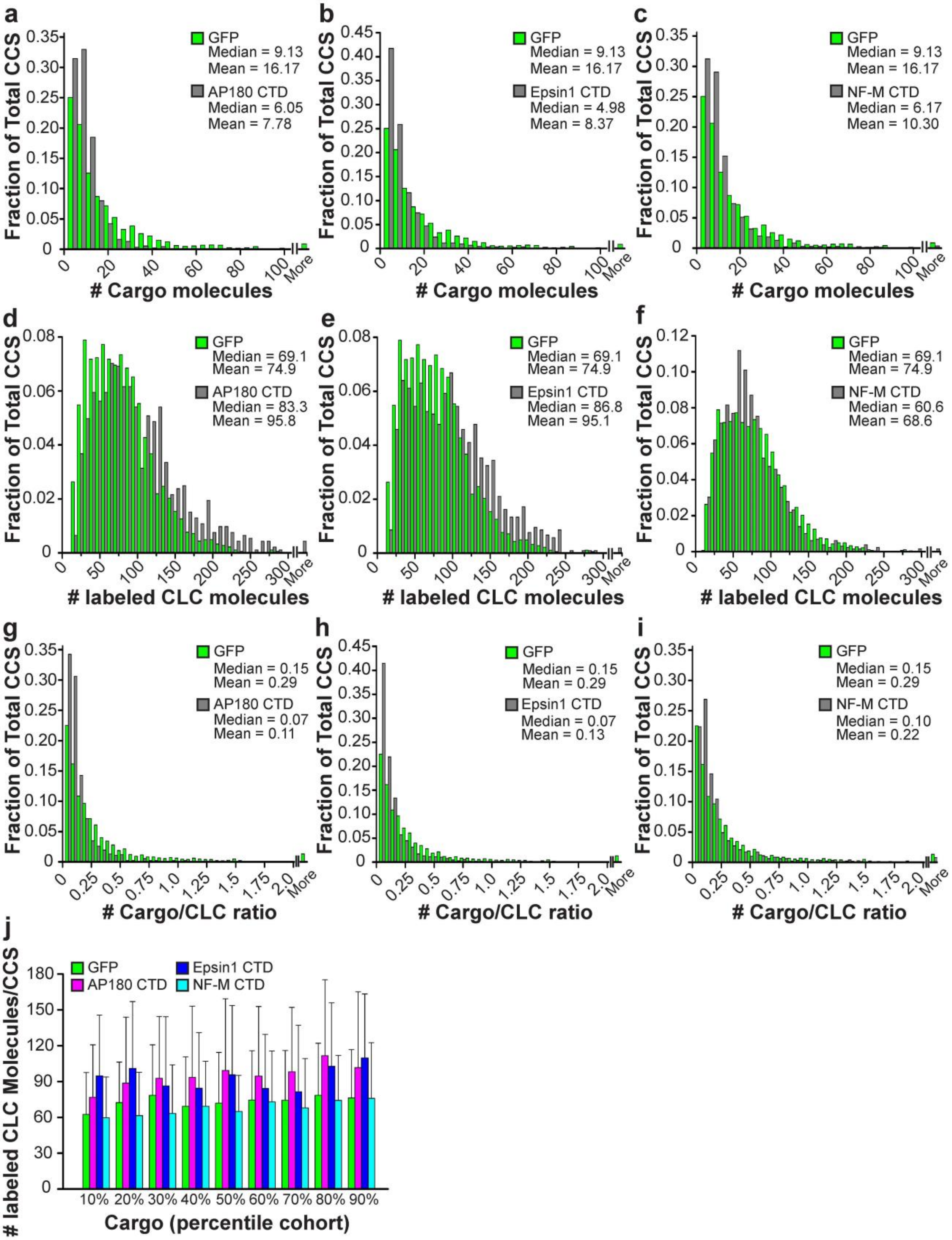

Supplementary Figure 6. Distribution of IDP Cargo and Clathrin levels within each clathrin-coated structure. Histograms representing the number of cargo molecules (GFP) per

clathrin-coated structure (CCS) (a-c) and labeled clathrin light chain (mCherry) molecules per structure (d-f). In all histograms green bars are the same data for cells expressing the GFP cargo and clathrin light chain (mCherry) as compared to cells expressing the IDP cargo. (a-c) Distribution of GFP cargo per CCS (green bars, mean ± s.d. = 16.2 ± 19.6 molecules) compared to (a) AP180 CTD cargo (gray bars, mean ± s.d. = 7.8 ± 7.2 molecules), (b) Epsin1 CTD cargo (gray bars, mean ± s.d. = 8.4 ± 10.0 molecules) and (c) NF-M CTD cargo (gray bars, mean ± s.d. = 10.3 ± 13.0 molecules). (d-f) Number of mCherry-labeled clathrin light chain molecules (mCherry) for cells expressing the GFP cargo (green bars, mean ± s.d. = 74.9 ± 42.4 molecules) compared to cells expressing (d) AP180 CTD cargo (gray bars, mean ± s.d. = 95.8 ± 57.5 molecules), (e) Epsin1 CTD cargo (gray bars, mean ± s.d. = 93.1 ± 53.3 molecules) and (f) NF-M (gray bars, mean ± s.d. = 68.6 ± 40.9 molecules). (g-i) Distribution of the ratio of GFP cargo molecules/CLC molecules per CCS (green bars, mean ± s.d. = 0.29 ± 1.7) compared to (g) AP180 CTD cargo (gray bars, mean ± s.d. = 0.11 ± 1.3), (h) Epsin1 CTD cargo (gray bars, mean ± s.d. = 0.13 ± 1.4) and (i) NF-M CTD cargo (gray bars, mean ± s.d. = 0.22 ± 1.4). (j) Number of mCherry labeled clathrin light chain molecules per CCS, displayed as percentile cohorts for each of the cargo constructs (GFP, green bars; AP180 CTD, magenta bars; Epsin1 CTD, blue bars; NF-M CTD, cyan bars). Experimental n values for all histograms are as follows: GFP, n = 1825 structures, 17 cells; AP180 CTD, 925 structures, 12 cells; Epsin1 CTD, n = 1047 structures, 9 cells; NF-M CTD, n = 1287 structures, 19 cells. All values reported are the mean ± s.d. Data was acquired from 2 dishes of cells per condition representing independent transfections.

Supplementary Methods

Membrane Coverage Measurements To measure the number of labeled proteins bound to the SLB surface per area for experiments measuring crowded diffusion rates, we executed the following steps: (i) determine the average brightness (number of photons per time) per single dye molecule on a sparsely covered surface, (ii) measure the average number of photons collected from the crowded membrane surface within the confocal spot size, (iii) divide this value by the average brightness of a single molecule to determine the number of molecules diffusing within the confocal spot size on the crowded membrane, (iv) measure the confocal spot size, (v) divide the number of molecules per confocal spot by the area of the confocal spot to arrive at a measure of the number of labeled molecules per membrane area on the crowded surface.

(i) Determine the brightness per single dye molecule: The calibrated single molecule fluorescence for Atto488 was determined by binding a dilute concentration of Atto488 labeled proteins (< 1 dye protein^{-1}) to the surface of an SLB. Collecting FCS data on protein diffusion over the membrane surface, the correlation amplitude provides a measure of the number of molecules diffusing within the laser focus. Then, a nanopositioning piezoelectric stage coupled with Becker and Hickl electronics for TCSPC was used to sample the fluorescence intensity (photons per time) within the laser focus at an array (32 x 32) of positions on the SLB surface, typically 200 nm apart. Dividing the average value from these measurements by the number of molecules within the laser focus (from the amplitude of the FCS correlation) provides a measure of the average brightness of individual fluorophores (photons per time).

(ii) Measure the total number of photons collected from the crowded membrane surface within the confocal spot size: A 2 µM solution of 95 parts dark protein: 5 parts Atto488 labeled protein was incubated with an SLB composed of POPC lipids with varying molar % of DOGS-Ni-NTA

lipids. ENTHΔH0, Epsin1 FL and AP180 CTD were bound to the SLB for 20 minutes (60 minutes for AP180 CTD) before being washed thoroughly to remove unbound protein. Then the brightness of the membrane surface (photons per time within the confocal spot) was measured as described in part i.

(iii) The brightness of the crowded membrane surface was divided by the average brightness of a single molecule to determine the number of labeled molecules diffusing within the confocal spot size. Since 5% of the membrane-bound molecules were labeled, the total density of membrane-bound molecules was calculated.

(iv) Measure the confocal spot size: The diameter of the laser focus was determined by collecting intensity scans of fluorescent beads of sub-diffraction limited size at 50 nm steps pixel^{-1}. A Gaussian function was fit to the pixel intensity profile of the beads. The standard deviations obtained from the Gaussian fits were averaged for all beads, and the diameter of the laser focus was defined as two standard deviations (~330 nm).

(v) Divide the number of molecules per confocal spot by the area of the confocal spot to arrive at a measure of the number of labeled molecules per crowded membrane area.

Experimental n values for photon counting scans are as follows: for ENTHΔH0, n = 11 scans, 1 SLB (1% DOGS-Ni-NTA); n = 10 scans, 1 SLB (2% DOGS-Ni-NTA); n = 10 scans, 1 SLB (5% DOGS-Ni-NTA); n = 12 scans, 1 SLB (7.5% DOGS-Ni-NTA); n = 41 scans, 4 SLBs (10% DOGS-Ni-NTA); for Epsin1 FL, n = 23 scans, 2 SLBs (1% DOGS-Ni-NTA); n = 21 scans, 2 SLBs (2% DOGS-Ni-NTA); n = 25 scans, 2 SLBs (3.5% DOGS-Ni-NTA); n = 21 scans, 2 SLBs (5% DOGS-Ni-NTA); for AP180 CTD, n = 25 scans, 2 SLBs (2% DOGS-Ni-NTA); n = 25 scans, 2 SLBs (3.5% DOGS-Ni-NTA); n = 20 scans, 2 SLBs (5% DOGS-Ni-NTA); n = 21 scans, 2 SLBs (10% DOGS-Ni-NTA).
